# Overview of the *CCP*4 suite and current developments

**DOI:** 10.1107/S0907444910045749

**Published:** 2011-03-18

**Authors:** Martyn D. Winn, Charles C. Ballard, Kevin D. Cowtan, Eleanor J. Dodson, Paul Emsley, Phil R. Evans, Ronan M. Keegan, Eugene B. Krissinel, Andrew G. W. Leslie, Airlie McCoy, Stuart J. McNicholas, Garib N. Murshudov, Navraj S. Pannu, Elizabeth A. Potterton, Harold R. Powell, Randy J. Read, Alexei Vagin, Keith S. Wilson

**Affiliations:** a STFC Daresbury Laboratory, Daresbury, Warrington WA4 4AD, England; b STFC Rutherford Appleton Laboratory, Chilton, Didcot OX11 0QX, England; c York Structural Biology Laboratory, Department of Chemistry, University of York, Heslington, York YO10 5YW, England; d Department of Biochemistry, University of Oxford, South Parks Road, Oxford OX1 3QU, England; e MRC Laboratory of Molecular Biology, Hills Road, Cambridge CB2 0QH, England; f University of Cambridge Department of Haematology, Cambridge Institute for Medical Research, Hills Road, Cambridge CB2 2XY, England; g Biophysical Structural Chemistry, Leiden University, PO Box 9502, 2300 RA Leiden, The Netherlands

**Keywords:** *CCP*4, macromolecular crystallography, software, collaboration, automation, macromolecular structure determination

## Abstract

The *CCP*4 (Collaborative Computational Project, Number 4) software suite is a collection of programs and associated data and software libraries which can be used for macromolecular structure determination by X-ray crystallography. The suite is designed to be flexible, allowing users a number of methods of achieving their aims. The programs are from a wide variety of sources but are connected by a common infrastructure provided by standard file formats, data objects and graphical interfaces. Structure solution by macromolecular crystallo­graphy is becoming increasingly automated and the *CCP*4 suite includes several automation pipelines. After giving a brief description of the evolution of *CCP*4 over the last 30 years, an overview of the current suite is given. While detailed descriptions are given in the accompanying articles, here it is shown how the individual programs contribute to a complete software package.

## Abstract 2

An overview of the *CCP*4 software suite for macromolecular crystallography is given.

## 1. Introduction

CCP4 (Collaborative Computational Project, Number 4, 1994 ▶) exists to produce and support a world-leading integrated suite of programs that allows researchers to determine macromolecular structures by X-ray crystallography and other bio­physical techniques. CCP4 aims to develop and support the development of cutting-edge approaches to the experimental determination and analysis of protein structure and to integrate these approaches into the *CCP*4 software suite. CCP4 is a community-based resource that supports the widest possible researcher community, embracing academic, not-for-profit and for-profit research. CCP4 aims to play a key role in the education and training of scientists in experimental structural biology. It encourages the wide dissemination of new ideas, techniques and practice.

In this article, we give an overview of the CCP4 project, past, present and future. We begin with a historical perspective on the growth of the software suite, followed by a summary of the current functionality in the suite. We then discuss ongoing plans for the next generation of the suite which is in development. In this account we focus on the suite as a whole, while other articles in this issue delve deeper into individual programs. We intend that this article could serve as a general literature citation for the use of the *CCP*4 software suite in structure determination, although we also encourage the citation of individual programs, many of the relevant references for which are included here. While we focus here on the *CCP*4 software suite, we would emphasize that comparable functionality is available in other software packages such as *SHARP*/*autoSHARP* (Vonrhein *et al.*, 2007 ▶), *SHELX* (Sheldrick, 2008 ▶), *ARP*/*wARP* (Langer *et al.*, 2008 ▶), *PHENIX* (Adams *et al.*, 2010 ▶) and many others.

## 2. Evolution of the *CCP*4 software suite

The *CCP*4 software suite is a collection of programs implementing specific algorithms concerned with macromolecular structure solution from X-ray diffraction data. Significantly, it is a collection of autonomous and independently developed programs. While some have been commissioned by the academic committees overseeing the CCP4 project, the majority originate from the community to address a perceived gap in current functionality or to implement newly developed algorithms. The result is a collection of around 200 programs, ranging from large programs which are effectively packages in themselves to small ‘jiffy’ programs. Over the years the suite has grown continuously, with each major release featuring significant new software (see Table 1 ▶). Unsurprisingly, there is overlap of functionality, with several programs performing a particular task, albeit often using different approaches. The question then is how to combine these programs into a software suite, both in terms of ensuring communication between the different programs and in helping both naïve and experienced users to navigate through the suite.

Early on in the history of *CCP*4, there was an agreement for all programs to use the same file formats for data files. Formats were specified for diffraction data (the LCF format, later replaced by the MTZ format) and for electron-density maps (the CCP4 map format), while for atomic coordinates the PDB format was adopted. A software library was developed to facilitate reading and writing of these data formats and thereby ensure standardization of the formats. Originally supporting only Fortran programs, the library was re-written to support both Fortran and C/C++ as well as scripting languages (Winn *et al.*, 2002 ▶). The CCP4 set of libraries has since expanded to cover a wider range of crystallographic tasks, in particular with the addition of the Clipper library (Cowtan, 2003 ▶), the MMDB library (Krissinel *et al.*, 2004 ▶) and the CCTBX library (Grosse-Kunstleve *et al.*, 2002 ▶) from the *PHENIX* project (Adams *et al.*, 2010 ▶).

**Figure 1:**
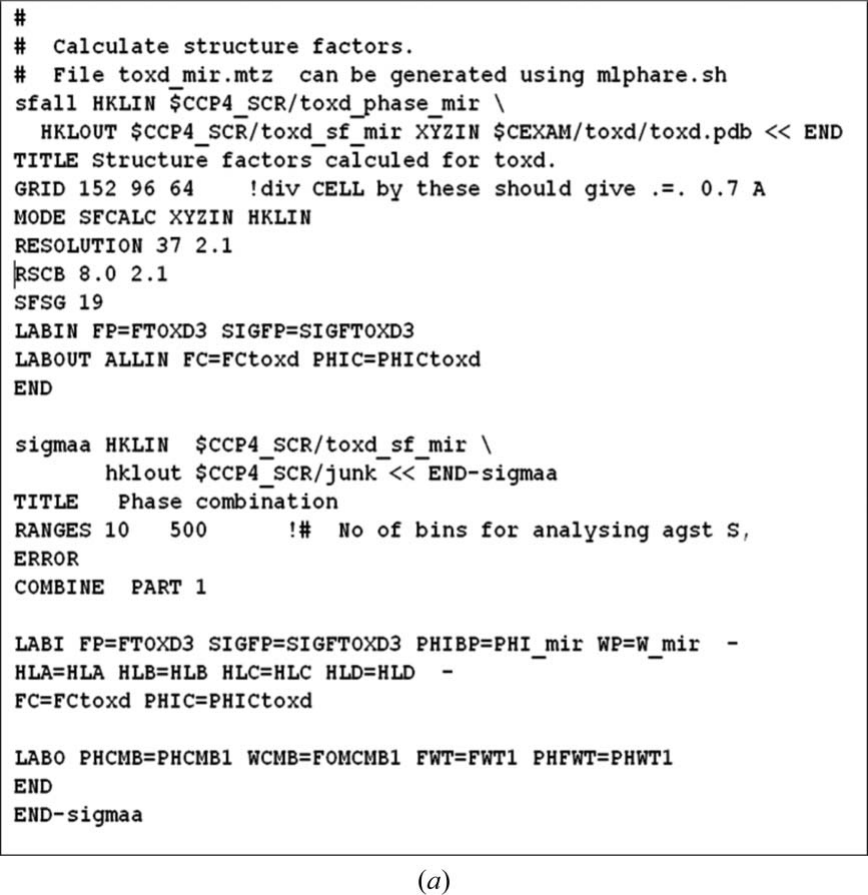

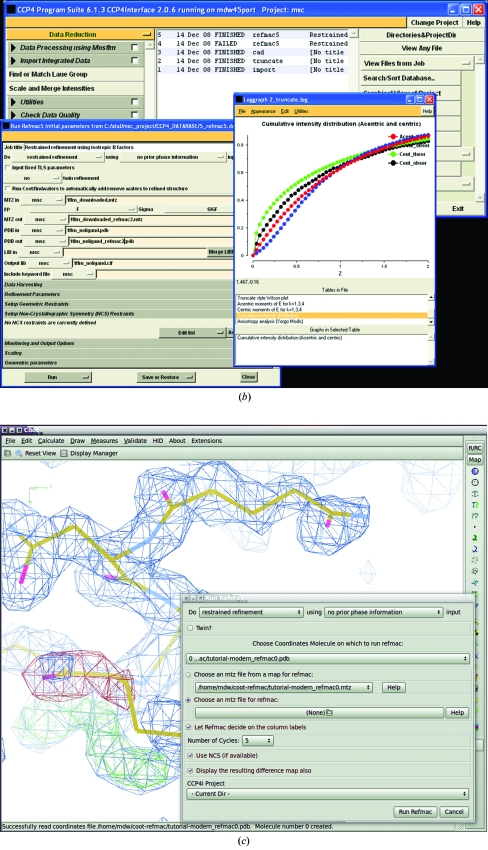
The changing face of *CCP*4: (*a*) a typical script chaining programs together, (*b*) the *ccp*4*i* graphical user interface and (*c*) the molecular-graphics viewer *Coot* (Emsley *et al.*, 2010 ▶) from which refinement and validation programs can be launched.

Crystallographic tasks were performed by writing or adapting scripts (*e.g.* Unix shell or VMS scripts) to link together a number of programs (Fig. 1*a* ▶) and the suite can still be run in this way. The programs communicate solely *via* the data files which are passed between them. The user sets program options based on the program documentation and the expected results from earlier steps. A major change was introduced in 2000 with the release of the graphical user interface *ccp*4*i* (Fig. 1*b* ▶; Potterton *et al.*, 2003 ▶). Task interfaces help the user to prepare run scripts. Details of how to run specific programs are largely hidden, as are the jiffy programs used to perform minor functions such as format conversion. Some limited intelligence in the interface code allows program options to be customized according to properties of the data and/or the desired objective. *ccp*4*i* interfaces are now available for all of the commonly used *CCP*4 programs as well as for several non-*CCP*4 programs (*e.g. ARP*/*wARP*; Langer *et al.*, 2008 ▶).

The *ccp*4*i* interface also introduced for the first time tools for helping the user to organize data. Jobs that have been run were recorded in a ‘database’ (in reality a directory of files) with tools to access and interpret the files saved there. Jobs are further organized into projects, representing different structure solutions. There are now plans to update the *CCP*4 GUI (see §[Sec sec4]4), but the impact of the original *ccp*4*i* on the suite should not be underestimated.

In the last few years, two other modes of accessing the *CCP*4 suite have emerged. On the one hand, the latest version of the suite contains four complementary automation pipelines, namely *xia*2 (Winter, 2010 ▶), *CRANK* (Ness *et al.*, 2004 ▶), *MrBUMP* (Keegan & Winn, 2007 ▶) and *BALBES* (Long *et al.*, 2008 ▶). These pipelines attempt to perform large sections of the full structure solution (*e.g.* phasing) without user intervention. This is achieved partly through the use of a large number of trials, trying different protocols and performing parameter scanning. Such an approach can be very powerful, using cheap computer power to make many more attempts than a user would manually. Automation pipelines have been realised in the last few years because of the maturity of the underlying programs and the availability of sufficient computer power to support multiple trials.

On the other hand, graphical programs for interactive use have become more powerful. Rather than simply reviewing the results of previously run programs and performing interactive model editing, *Coot* (Emsley *et al.*, 2010 ▶) can launch separate refinement and validation programs (Fig. 1*c* ▶). Similarly, *iMOSFLM* can be used to interface the data-processing programs *POINTLESS* and *SCALA*. In some ways this is a completely different scenario to the automation pipelines. User interaction is paramount, with crystallo­graphy programs acting as tools to be invoked. The user can become familiar with the data and structure and use this to make intelligent decisions. Such an approach has also become possible because of the maturity of the invoked programs and the availability of sufficient computer power to run the programs interactively.

## 3. Overview of current functionality

In this section, we give an overview of the current functionality of the *CCP*4 software suite (corresponding to release series 6.1 at the time of writing). We summarize the automation pipelines and individual programs included in the suite; many more details can be found in the accompanying articles in this issue. We present the functionality in the traditional manner, starting at data processing and ending at validation. However, it is becoming increasingly apparent that these neat categories are breaking down.

### 3.1. Data processing

**Figure 2:**
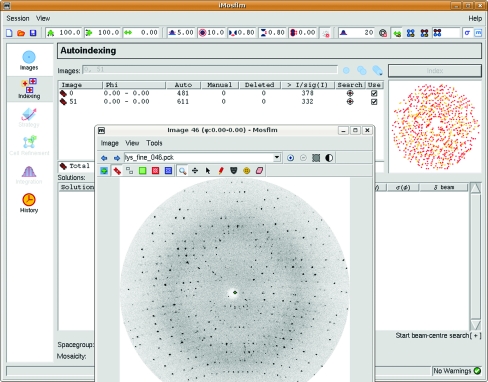
The *iMOSFLM* interface, showing the main window and a display of one selected image.

The earliest starting point for entry into the *CCP*4 suite is a set of X-ray diffraction images. The data-reduction program *MOSFLM* (Leslie, 2006 ▶) will take a set of diffraction images, identify spots on each image, index the diffraction pattern and thus identify the Bragg peaks, and integrate the spots. The output is a list of integrated intensities and their standard uncertainties labelled by the *h*, *k*, *l* indices. Associated information includes the batch number of the image from which the intensity was obtained, whether the peak was full or partial and the symmetry operation that relates the particular observation to the chosen asymmetric unit. *MOSFLM* continues to be improved, with support added recently for Pilatus detectors, addition of automatic backstop masking *etc*. The most visible change is the replacement of the old X-­windows-based interface with the Tcl-based *iMOSFLM* interface (Fig. 2 ▶), which guides the user in a stepwise manner through the stages of data processing.

*POINTLESS* is a relatively new program whose primary purpose is to identify the Laue group of a crystal from an unmerged data set (Evans, 2006 ▶). The program will also attempt to identify the space group from an analysis of systematic absences. A secondary purpose is to test the choice of indexing and re-index a data set if necessary.

Given a choice of space group, the program *SCALA* (Evans, 2006 ▶) will refine the parameters of a scaling function for an unmerged data set, apply scales to each observation of a reflection and merge all observations of a reflection to give an average intensity. It will also provide an improved estimate of the standard uncertainty of each intensity. The new program *CTRUNCATE* (which replaces the older *TRUNCATE*; Stein, unpublished program) can then convert the intensities to structure-factor amplitudes, although downstream programs increasingly use the mean intensities directly. Perhaps more importantly, *CTRUNCATE* will analyse a data set for signs of twinning, translational noncrystallographic symmetry (NCS), anisotropy and other notable features, since it is best to identify problems before attempting phasing. The program *SFCHECK* (Vaguine *et al.*, 1999 ▶) will also provide an analysis of a data set, including testing for twinning and translational NCS, estimating the optical resolution and the anisotropy, and plotting the radial and angular completeness.

The previous steps of data processing are automated by the *xia*2 pipeline (Winter, 2010 ▶). From a directory of images, *xia*2 will identify the type of experiment (multi-wedge, multi-pass, multi-wavelength) and process accordingly. The pipeline will determine the point group, space group and correct indexing. Multiple processing pipelines using alternative underlying programs are supported. At the end, the user should have a set of merged structure-factor amplitudes suitable for input to phasing.

### 3.2. Experimental phasing

*CCP*4 includes the *CRANK* pipeline (Ness *et al.*, 2004 ▶), which covers experimental phasing and beyond, and interfaces with several *CCP*4 and non-*CCP*4 programs. Heavy-atom sub­structure detection is performed by *AFRO*/*CRUNCH*2 (de Graaff *et al.*, 2001 ▶) or by *SHELXC*/*D* (Sheldrick, 2008 ▶) and initial phasing is carried out by *BP*3 (Pannu *et al.*, 2003 ▶; Pannu & Read, 2004 ▶) or *SHELXE* (Sheldrick, 2008 ▶). Phase improvement is carried out by *SOLOMON* (Abrahams & Leslie, 1996 ▶), *DM* (Cowtan *et al.*, 2001 ▶) or *Pirate* (Cowtan, 2000 ▶) and automated model building by *Buccaneer* (Cowtan, 2006 ▶; Cowtan, 2008 ▶) or *ARP*/*wARP* (Langer *et al.*, 2008 ▶). *CRANK* thus supports a range of underlying software handling the communication of data and allowing the user to trial different combinations.

*CCP*4 includes a number of additional individual programs, each of which has its own particular strength. The long-standing *CCP*4 program *MLPHARE* for phasing still works in straight­forward cases and is fast to use. *ACORN* (Jia-xing *et al.*, 2005 ▶; Dodson & Woolfson, 2009 ▶) uses *ab initio* methods for the determination of phases starting from a small fragment which could be a single heavy atom. The use of *ab initio* methods usually requires atomic resolution data, since it assumes atomicity of the electron density. However, a variant of the so-called free-­lunch algorithm (Jia-xing *et al.*, 2005 ▶) allows the temporary generation of phases to atomic resolution which the *ACORN* method can utilize. The *OASIS* program (Wu *et al.*, 2009 ▶) also uses *ab initio* methods to break the phase ambiguity in SAD/SIR phasing.

*Phaser* (McCoy *et al.*, 2007 ▶) can obtain phase estimates starting from known heavy-atom positions and SAD data. Log-likelihood gradient (LLG) maps are used to automatically find additional sites for anomalous scatterers and to detect anisotropy in existing anomalous scatterers. *Phaser* can also use a partial model, for example from a molecular-replacement solution that is hard to refine, as a source of phase information to help locate weak anomalous scatterers and thus improved phases. The latter reflects the view of experimental phasing and molecular replacement as just two sources of phase information rather than two separate techniques.

### 3.3. Molecular replacement

*CCP*4 includes two pipelines for molecular replacement (MR): *MrBUMP* (Keegan & Winn, 2007 ▶) and *BALBES* (Long *et al.*, 2008 ▶). Both start from processed data and a target sequence and aim to deliver a molecular-replacement solution consisting of positioned and partially refined models. *BALBES* uses its own database of protein molecules and domains taken from the PDB and customized for MR, while *MrBUMP* uses public databases and a set of widely available bioinformatics tools to generate possible search models.

*BALBES* is based around the MR program *MOLREP* (Vagin & Teplyakov, 1997 ▶, 2010 ▶), while *MrBUMP* can also use the program *Phaser* (McCoy *et al.*, 2007 ▶). Both *MOLREP* and *Phaser* are also available as stand-alone programs in *CCP*4. As well as providing rotation and translation functions, whereby a search model is positioned in the unit cell to give an initial estimate of the phases, these programs provide additional functionality, including a significant contribution to automated decision-making. For instance, a single run of *Phaser* can search for several copies each of several components in the structure of a complex, testing different possible search orders and trying different possible choices of space group.

The search model for MR may be an ensemble of structures, a set of models from an NMR structure or an electron-density map. Phases for the target may be available, so that the search model is to be fitted into electron density, or there may be density available from an electron-microscopy experiment. The MR step can be followed by rigid-body refinement and the packing of the MR solution can be checked. Much of this functionality is common to *Phaser* and *MOLREP*, but there are a number of differences in implementation, so that both may prove useful in certain circumstances.

A crucial component of MR is the selection and preparation of search models. The program *CHAINSAW* (Stein, 2008 ▶) takes as input a sequence alignment which relates residues in the search model to residues in the target protein and uses this information to edit the search model appropriately. The output model is labelled according to the target sequence. *MOLREP* (Lebedev *et al.*, 2008 ▶) can take as input the target sequence and performs its own alignment to the search model in order to edit the search model.

### 3.4. Phase improvement and automated model building

Having obtained initial phases from experimental phasing, the next step is phase improvement (density modification) to give a map that can be built into. When phases come from molecular replacement, phase improvement may also be useful to reduce model bias. For a long time, the main *CCP*4 phase-improvement programs were *DM* (Cowtan *et al.*, 2001 ▶) and *SOLOMON* (Abrahams & Leslie, 1996 ▶), which covered the standard techniques of solvent flattening/flipping, histogram matching and NCS averaging. More recently, statistically based methods have been incorporated into the program *Pirate* (Cowtan, 2000 ▶). *Pirate* can give better results, but has been found to be inconveniently slow. The latest program *Parrot* (Cowtan, 2010 ▶) achieves similar improvements but is also fast and automated.

Given an electron-density map, automated model building is provided in *CCP*4 by *Buccaneer* (Cowtan, 2006 ▶, 2008 ▶). This finds candidate C^α^ positions, builds these into chain fragments, joins the fragments together and docks a sequence. NCS can be used to rebuild and complete related chains. Since version 1.4, there is support for model (re)building after molecular replacement and for supplying known structural elements such as heavy atoms. The *CCP*4 suite includes an interface for alternating cycles of model building with *Buccaneer* with cycles of model refinement with *REFMAC*5. The supplementary program *Sloop* (Cowtan, unpublished program) builds missing loops using fragments taken from the Richardson’s Top500 library of structures (Lovell *et al.*, 2003 ▶) to fill gaps in the chain. The chance of finding a good fit falls with increasing size of the gap, but the method may work for loops of up to eight residues in length.

*RAPPER* (Furnham *et al.*, 2006 ▶) provides a conformational search algorithm for protein modelling, which can produce an ensemble of models satisfying a wide variety of restraint information. In the context of *CCP*4, restraints on the modelling are provided by the electron density and/or the locations of the C^α^ atoms. The *ccp*4*i* interface includes modes for loop building or for building the entire structure.

### 3.5. Refinement and model completion

The aim of macromolecular crystallography is to produce a model of the macromolecule of interest which explains the diffraction images as accurately and completely as possible. Both the form of the model and the parameters of the model need to be defined. Refinement is the process of optimizing the values of the model parameters and in *CCP*4 is performed by the program *REFMAC*5 (Murshudov *et al.*, 1997 ▶). *REFMAC*5 will refine atomic coordinates and atomic isotropic or anisotropic displacement parameters (Murshudov *et al.*, 1999 ▶), as well as group parameters for rigid-body refinement and TLS refinement (Winn *et al.*, 2001 ▶, 2003 ▶). It will also refine scaling parameters and a mask-based bulk-solvent correction.

When good-quality experimental phases are available, these can be included as additional data (Pannu *et al.*, 1998 ▶). More recently, it has become possible to refine directly against anomalous data for the cases of SAD (Skubák *et al.*, 2004 ▶) and SIRAS (Skubák *et al.*, 2009 ▶) without the need for estimated phases and phase probabilities. *REFMAC*5 will also now refine against twinned data (Lebedev *et al.*, 2006 ▶), automatically recognising the twin laws and estimating the corresponding twin fractions.

The nonprotein contents of the crystal are often of most interest, such as bound ligands, cofactors, metal sites *etc*. Correct refinement at moderate or low resolution requires a knowledge of the ideal geometry together with associated uncertainties. In *REFMAC*5 this is handled through a dictionary of possible ligands (Vagin *et al.*, 2004 ▶), with details held in mmCIF format. Dictionary files can be created through the tools *SKETCHER* and *JLIGAND*.

Refinement goes hand-in-hand with rounds of model building which add/subtract parts of the model and apply large structural changes that are beyond the reach of refinement. In addition to the automated procedures of *Buccaneer* and *RAPPER* described above, there are many model-building tools in *Coot* (Emsley *et al.*, 2010 ▶). A *ccp*4*i* interface to the popular *ARP*/*wARP* model-building package (Langer *et al.*, 2008 ▶) has also been available for many years.

### 3.6. Validation, deposition and publication

Validation is the process of ensuring that all aspects of the model are supported by the diffraction data, as well as con­forming with known features of protein chemistry. Although validation has traditionally been viewed as something that is performed at the end of structure determination, just before deposition, it is now appreciated that validation is an integral part of the process of structure solution, which should be carried out continually. *CCP*4 includes a wide variety of validation tools, all of which should be run to gain a complete picture of model quality. *Coot* (Emsley *et al.*, 2010 ▶) has a dedicated drop-down menu of validation tools which can and should be applied as the model is being built. *Coot* can also extract warnings about particular links or outliers from a *REFMAC*5 log file. Warnings associated with specific atoms or residues are linked directly to the model as viewed in *Coot*.

The *ccp*4*i* ‘Validation and Deposition’ module contains further validation tools. As mentioned above, *SFCHECK* (Vaguine *et al.*, 1999 ▶) provides a number of measures of data quality, but if a model is provided it will also assess the agreement of the model with the data. *Sequins* (Cowtan, unpublished program) validates the assigned sequence against electron density (generated from experimental phases or from phases calculated from a side-chain omit process) and warns of mis­placed side chains or register errors. *RAMPAGE* (which is part of the *RAPPER* package; Furnham *et al.*, 2006 ▶) provides Ramachandran plots based on updated ϕ–ψ propensities. *PROCHECK* is also included, although the Ramachandran plots are no longer generated, having been superseded by *RAMPAGE*. *R*500 (Henrick, unpublished program) checks the stereochemistry in a given PDB file against expected values and lists outliers in REMARK 500 records.

The quaternary structure of the protein can be analysed with *PISA* (Krissinel & Henrick, 2007 ▶). This considers all possible interfaces in the crystal structure, estimates the free energy of dissociation, taking into account solvation and entropy effects, and predicts which interfaces are likely to be of biological significance.

**Figure 3:**
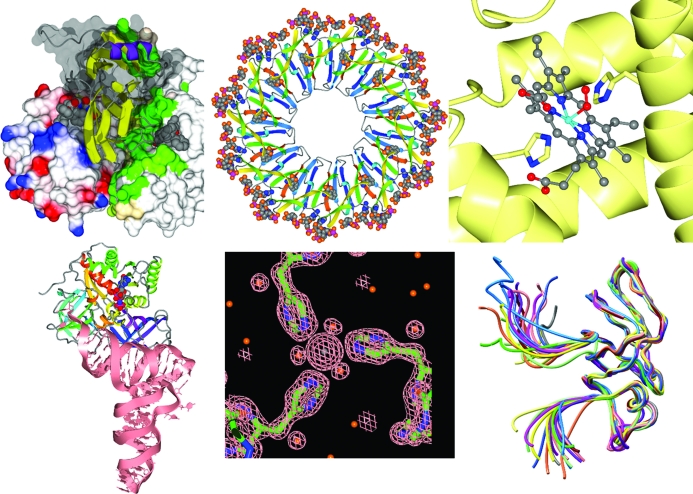
A montage of images from the molecular-graphics program *CCP*4*mg* (Potterton *et al.*, 2004 ▶).

The *CCP*4 molecular-graphics program *CCP*4*mg* (Potterton *et al.*, 2002 ▶, 2004 ▶) provides a simple means of generating publication-quality images and movies. As well as displaying coordinates in a wide variety of styles, *CCP*4*mg* can display molecular surfaces, electron density, arbitrary vectors and labels. The latest versions are built on the *Qt* toolkit, giving an enhanced look and feel (Fig. 3 ▶). Structures and views can be transferred between *CCP*4*mg* and *Coot*.

### 3.7. Jiffies and utilities

In addition to the main functionality described above, the *CCP*4 suite contains a large number of utilities for performing format conversions and various analyses. Reflection data processed in other software packages can be imported with the utilities *COMBAT*, *POINTLESS*, *SCALEPACK*2*MTZ*, *DTREK*2*SCALA* and *DTREK*2*MTZ*, while data can be exchanged with other structure-solution packages with *CONVERT*2*MTZ*, *F*2*MTZ*, *CIF*2*MTZ*, *MTZ*2*VARIOUS* and *MTZ*2*CIF*. There are several useful utilities based on the Clipper library (Cowtan, 2003 ▶), such as *CPHASEMATCH*, which will compare two phase sets and look for changes in origin or hand. There are also many useful utilities for analysing coordinate files. New programs based on the MMDB library (Krissinel *et al.*, 2004 ▶) include *NCONT* for listing atom contacts and *PDB_MERGE* for combining two PDB files.

## 4. Future plans

At the heart of the *CCP*4 suite are the set of algorithms encoded in individual programs. As always, we include new programs in each major release of the suite and will continue to do so. Since the source of novel software is usually independent developers, the additions to the suite are not centrally planned. Nevertheless, some current themes are clearly recognisable, such as automated model building, in particular for low-resolution data.

*CCP*4 also aims to enhance its functionality related to the maintenance and use of data on small molecules (ligands). Firstly, a considerably larger library of chemical compounds will be provided with the suite. Extended search functions will be provided to allow the efficient retrieval of known com­pounds or their close analogues. Secondly, existing functions for generating restraint data for new ligands will be enhanced by the inclusion of relevant software such as *PRODRG* (Schüttelkopf & van Aalten, 2004 ▶) into the suite, as well as by the development of new methods for structure reconstruction on the basis of partial similarity to structures in the library. Functionality will be available through a graphical front-end application, *JLIGAND*.

In addition to the core programs, the infrastructure of *CCP*4 continues to evolve to support the latest working practices. The current *CCP*4 GUI, *ccp*4*i*, was a major innovation and has served us well for over ten years (Potterton *et al.*, 2003 ▶). While it continues to provide a useful interface to the *CCP*4 suite, there are increasing demands from automation pipelines and users alike. In particular, there is a requirement to provide help on what to try next, advice which can be useful to both scientists and automated software. This depends on a robust assessment of the experimental data and the results of previous processing, which in turn requires good data management. We aim to address these issues through the development of a next-generation *CCP*4 interface.

There will also be changes in the way that *CCP*4 is delivered to the end user. We have all become used to automated up­dates to the software we use (*e.g.* Windows Update, Synaptic for Debian-based Linux or application-specific updates such as for Firefox). Some *CCP*4 programs do alert the users to the availability of newer versions and *CCP*4*mg* (Potterton *et al.*, 2002 ▶, 2004 ▶) will update the version on request. A *CCP*4-wide update mechanism is more difficult given the heterogeneous nature of the suite, but efforts in this direction are under way. A specific example of a remotely maintained crystallography platform is given by the US-based SBGrid Consortium.

The *CCP*4 suite is downloaded to a user’s machine or a local server before being run. This is in contrast to many biology software tools, which are web-based. Reasons for running *CCP*4 locally include the wallclock time of jobs, the detailed control required and the size of data files. Nevertheless, there is increasing usage of web servers for crystallographic tasks. A server at York (http://www.ysbl.york.ac.uk/YSBLPrograms/index.jsp) runs a number of *CCP*4 programs, including *BALBES* and *Buccaneer*, while *CCP*4 programs are included in a number of other services, for example the *ARP*/*wARP* server at Hamburg (http://cluster.embl-hamburg.de/ARPwARP/remote-http.html). Plans are under way to make more *CCP*4 functionality available *via* the web.

Finally, the coming years will see increasing integration of crystallography with other techniques, both experimental and theoretical. *CCP*4 aims to contribute towards efforts, such as the European infrastructure project INSTRUCT, to ease the transfer of data to and from these other domains.

## Figures and Tables

**Table 1:** A summary of major *CCP*4 releases within the last ten years

Major release	Date	No. of binaries	Highlights
6.1	December 2008	238	Automation pipelines
6.0	February 2006	212	*BP*3, *Phaser*, *Pirate*
5.0	May 2004	180	C-based libraries
4.2	April 2002	177	*ACORN*, *BEAST*, *PROFESSS*
4.1	January 2001	172	*REFMAC*5
